# Northern populations of Finnish raccoon dogs are active at the range edge and unhindered by movement boundaries

**DOI:** 10.1186/s40462-025-00601-1

**Published:** 2025-11-12

**Authors:** Purabi Deshpande, Pyry Toivonen, Vesa Selonen

**Affiliations:** 1 Department of Biology, University of Turku, Turku, FI-20014 Finland; 2 Natural Resources Institute Finland, Turku, FI-20520 Finland

**Keywords:** Dispersal, Invasion front, Invasive species, Mesopredators, Movement ecology, Nyctereutes procyonoides, Resource selection functions

## Abstract

Studying movements of invasive species is important for controlling their spread into new areas and understanding how their populations can be controlled in the regions where they have already spread. Additionally, animals at the range edge are known to behave differently from those at the range core. Such differences in movement behaviour can further affect the spread of a species into new areas. Here, we use data from GPS tracking of 103 individuals of the invasive raccoon dog (*Nyctereutes procyonoides*) in Finland to examine the movement of the species. First, we determine whether movement distances differ in the range edge and range core. Next, focusing on dispersing individuals, we explore whether their movements are directional. Finally, we investigate whether the dispersal by raccoon dogs is affected by movement boundaries, such as waterbodies and roads. We show that raccoon dogs at the range edge move larger distances than those in the range core. The dispersing individuals do not show a preference to moving in any particular direction. Finally, we do not find evidence for waterbodies and roads acting as movement boundaries for raccoon dogs. Our results indicate that raccoon dogs are very active at the range edge and highlight how the movement distances of a species might be underestimated if individuals from the invasion front are not studied. The raccoon dog’s spread is likely to continue into favourable habitats and not be hindered by roads or water bodies putting areas at higher latitudes and surrounding countries at the risk of invasion.

**Supplementary Information:**

The online version contains supplementary material available at 10.1186/s40462-025-00601-1.

## Background

Movement in the form of dispersal is a key event in the life history of many species [1]. Moving further from one’s home range ensures mixing of the gene pool, which is advantageous to the survival of the species, for example, by avoiding problems caused by inbreeding [2]. Dispersal also enhances invasion of new living areas, thus being an important process behind the distribution of the species [3]. Currently, anthropogenic climate change and other human activities are forcing some species to move away from their historic home ranges while simultaneously creating potential living areas for others [4]. The latter is particularly the case for introduced species which can on occasion become harmful invasive species with negative effects on native ecosystems [4, 5].

Understanding dispersal and movement behavior of invasive species provides us with knowledge on its colonization potential. For example, the dispersal distances and barriers for movement occurring in the landscape determine the areas within the potential reach of the moving individuals [1]. The barriers to the movement of terrestrial animals can be human made, such as roads, or natural, such as water bodies. For example, smaller mammals like rodents may avoid crossing even smaller roads [6], but larger animals like caribou (*Rangifer tarandus*) [7] and wolves (*Canis lupus*) are seen to use roads and other linear human made structures to move [8]. Similar to roads, waterbodies like rivers and lakes can affect the movement patterns of dispersing animals [9, 10]. In general, it is clear that landscape structure affects patterns of movements of animals [11–13], but dispersing individuals may be well motivated for moving long distances, crossing barriers and showing directional movement patterns [1, 14, 15].

Earlier research on species that are expanding their ranges has shown that individuals within the range core and those at the range edge can express different movement activity. For example, in the case of the invasive cane toads in Australia (*Rhinella marina*) individuals at the invasion front show higher long-term dispersal rates [16] and high directionality in movement [17]. Additionally, individuals at the invasion front could be under selection to be more active and move longer distances [18] or on the other hand, in certain cases like invasive bank voles (*Myodes glareolus*) in Ireland, individuals at the range front can also be more risk averse which might decrease the movement distances [19]. Hence, studying movements of animals both at the range core and at the range edge is important to understand the species colonization potential. This is essential for predicting the possible future scenarios for the species in the face of climate change, for both native species moving into new areas and introduced species that can become harmful invasives potentially negatively affecting local ecosystems [16].

In this study, we explore the dispersal and movements of adult raccoon dogs (*Nyctereutes procyonoides*) in Finland. The raccoon dog is a medium sized omnivorous canid predator that weighs typically 5 kg in early summer and, in Finland, about 9 kg in autumn before winter sleep [20]. It is monogamous, dens in pairs and can produce a litter of up to nine offspring each year, starting at the age of one year. Raccoon dogs invaded Finland from the former Soviet Union in the 1950s [21]. Its current range covers most of the country except for the northern parts of Lapland, but the species is increasing in numbers near these areas [22]. Raccoon dogs are recognised harmful invasive species in Europe [21]. They harm native biodiversity in the form of being nest predators of ground nesting birds [23, 24], and in wetlands by preying on amphibians [25]. The species also has management strategies in place to control their populations [26, 27].

The raccoon dog’s European range is expanding, mainly consisting of Eastern and Northern Europe, Germany and Denmark [21]. The Finnish populations of raccoon dogs represent the northern most populations of the species. Hence understanding their possible expansion further poleward is essential knowledge of biogeography of the species. Additionally, the raccoon dog is still seen in low numbers in Sweden and Norway. Understanding their movement patterns and the barriers to their movement in Finland would provide crucial information to ensure that raccoon dogs from Finland do not spread into new regions in these neighbouring countries. Studying the behaviour of raccoon dogs at the range edge in Finland is also important as northern Finland has unique boreal habitats. These boreal ecosystems are already under immense stress as the arctic is warming faster than other parts of the world [28].

Here, we have compared (a) the displacement and distances covered by GPS-tracked adult raccoon dogs in Southern Finland (range core) with individuals in Northern Finland (range edge). (b) Next, for the individuals classified as dispersing, we explored in detail the direction of movements and speed of the movements in the dispersal phase and stationary phase. We defined dispersal as movements by adult individuals that were not within a territory but over larger distances, into new regions. (c) Finally, we investigated whether movement boundaries like roads and shorelines of waterbodies shape the dispersal distances and routes taken by the raccoon dogs. To maximize colonization potential, the range edge individuals should show longer dispersal movements than the range core individuals and have directional movements enhancing invasion at the range edge. However, the movement boundaries could decrease the invasion potential of the dispersing raccoon dogs by an increase in angles between steps and preventing directed movements.

## Methods

### Study area

**Fig. 1:**
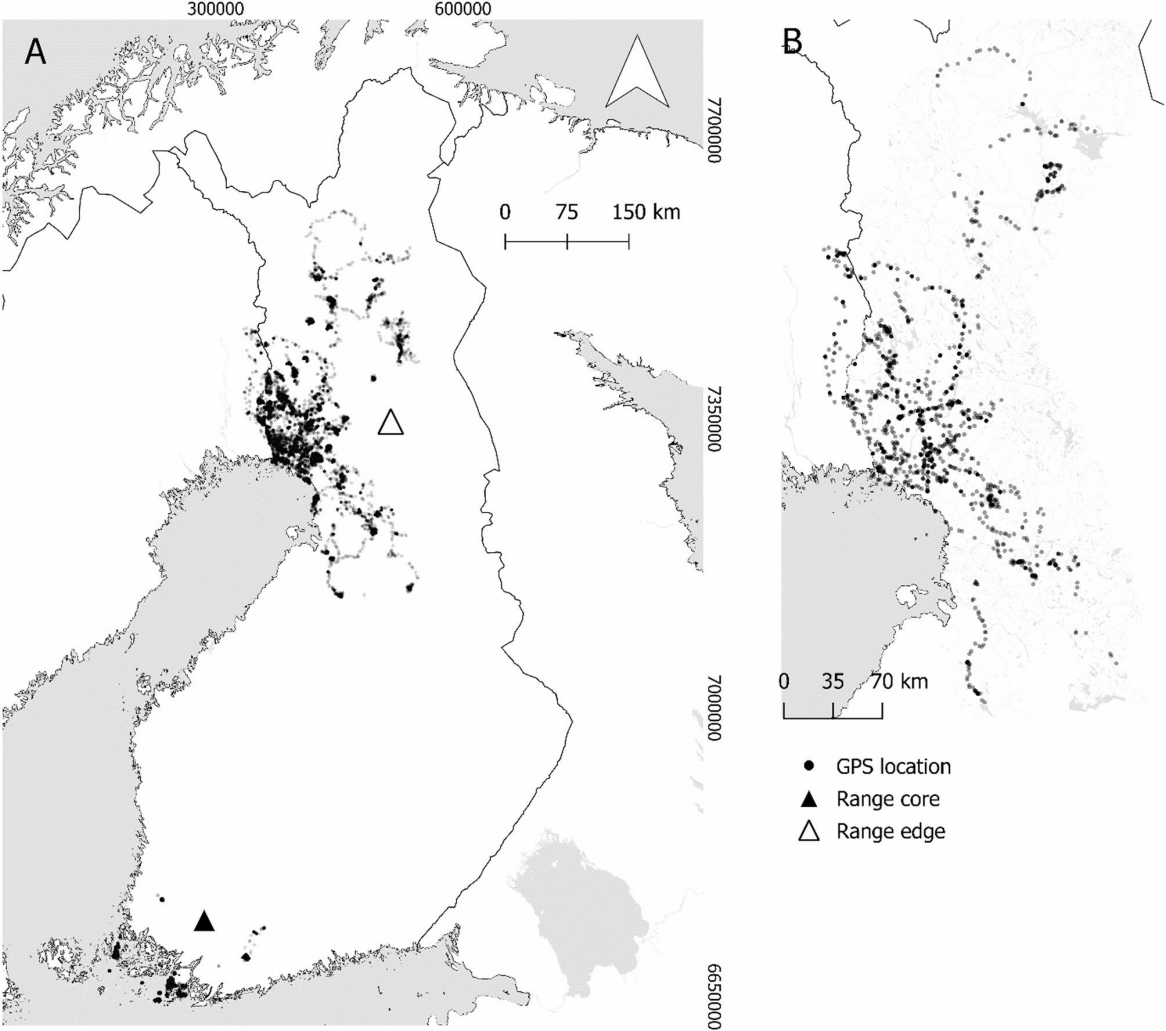
Map of raccoon dog movements. (**A**) GPS data from the range core and range edge and (**B**) the steps classified as dispersing. Location of each GPS location is marked with a transparent grey point; hence dark black regions indicate the individual moving more in a concentrated area. The coordinates on the map are in Finnish EUREF-FIN / TM35FIN(E, N) coordinate reference system (EPSG:3067). Additional polygon layers are produced by the National Land Survey of Finland and the Finnish Environment Institute

This study was carried out in a landscape of boreal managed coniferous and mixed forests [main tree species being the Scots pine (*Pinus sylvestris*), the Norway spruce (*Picea abies*) and the birch (*Betula spp.*)]. The forest landscape is fragmented by water bodies and agricultural areas. Agricultural areas and urban areas are found more in southern and southwestern Finland but occur sparsely within the rest of the country. The GPS-data originates from two areas in southern and northern Finland. The northern study area is located near Rovaniemi and Tornio (62 individuals) at the invasion front near the Arctic Circle (Fig. 1). The southern study area is located around Kemiö and Lohja (41 individuals). The Lohja area is inland, but Kemiö area consists of coastal mainland areas and islands of various sizes.

### Details about raccoon dog GPS data

Raccoon dogs were tracked with Followit Ultra-Light GSM/GPRS GPS collars that weighed 211 g, which is less than 5% of the weight of the animal. Tracking in southern study area was designed and executed by SLHSY (a coastal conservation and management NGO) in collaboration with Metsähallitus and the Finnish Wildlife Agency [29]. Tracking in northern study area was executed by the Finnish Wildlife Agency. Individuals were tracked mostly for the purpose of finding their dens, cubs, and partners. As a result of this the raccoon dogs experienced partner removals. The partner removal tends to increase raccoon dog movements [29, 30]. Unfortunately, due to lack of data we could not consider partner removal in the current study, but both of our study areas (range edge and range core) experienced similar levels of partner removal in terms of number of people participating in the removal. In addition, our previous analysis with a subset of individuals did not reveal effects on movement distances that would affect the conclusions of the current study [30]. At the range edge, on average, individuals were tracked for 569.9 ± 428.2 GPS locations while in the range core they were tracked for 539.3 ± 337.6 GPS location. The tracking at the range edge lasted on average for 145.2 ± 103.8 days and 159.7 ± 123.2 days in the range core. Tracking periods for each individual are detailed in Supplementary materials, Table 1.

### Preparing the GPS data

For calculating the displacement and distance travelled by raccoon dogs at the range edge and the range core we created tracks from the GPS locations. To get rid of locations made before and after collaring the animal (e.g. accidental locations during transport, final field testing etc.) we first filtered out the first and last three steps (i.e. first and last four GPS locations), or in the case of individuals used in the dispersal models, the whole first day of the designated tracking period was removed if it were more than the three steps. We also removed outlier steps that likely were errors in GPS signal where the raccoon dogs speed was clearly higher than 40 kmph, which is the highest speed that they are known to move at [31]. We also removed steps that spanned across more than a week. These were the gaps in the GPS-data from coldest periods of winter when raccoon dog stayed in nest in winter sleep [30].

The data consisted of individuals with variable sampling intervals between 2 and 4 h and individuals with regular three-hour sampling intervals. The assumption of integrated step-selection analysis (see below) is that animals are observed at regular time intervals [32]. Thus, for this analysis, we imputated all the individuals to a regular three-hour sampling interval using ‘momentuHMM’ package in R [33].

The GPS tracking data spanned over a period of more than a decade from 2011 to 2022 and the individuals varied in the time they were tracked. We had data on 41 individuals from the range core and 62 individuals from the range edge (Fig. 1A). After conducting classification to dispersal (see below), we found that 1 out of 41 individuals in range core and 35 out of 62 individuals in range edge were classified as dispersing individuals (Fig. 1B). After scrutinizing the data, 22 individuals in the range edge had sufficient data for analysing dispersal direction and responses to movement boundaries.

### Statistical methods

All the analyses were carried out in R version 4.4.1 [34].

#### Displacement and distance covered by raccoon dogs at range core and range edge

We used the ‘amt’ package to calculate the distances covered by raccoon dogs from the GPS data using the ‘make_track’ and ‘steps’ functions [35]. The step lengths were then added for each individual to calculate the total distances covered by each raccoon dog. We also used these steps to calculate the distances covered (sum of all steps) by the range edge and range core individuals during different months of the year. Next, by taking the coordinates for the first and last step of an individual we calculated the displacement (distance between first and last location) of individuals using the ‘st_distance’ function of the ‘sf’ package [36]. Finally, we used a t-test to determine if the distances travelled and displacement by individuals in the range core was different from the individuals at the range edge.

#### Classification of movements to dispersal and determining direction and speed of dispersal

We focused on the individuals at the range edge for this part of the analysis as they were seen to be moving further as compared to the individuals at the range core. We tried using automated or model-driven methods [MigrateR package [37] and Smoove [38] to classify steps to dispersal movement but found these unreliable as they did not work with all of the data (producing error messages) and/or they classified movement that was not clearly dispersal, e.g. movement from one edge of location clusters (home range) to other edge. The home ranges in north are large [39], which complicated the use of these methods. Hence, we relied on manual classification of dispersal by first using the net squared displacement over time (NSD plots) for each individual (Supplementary materials, Figs. 1 and 4) and then checked with the coordinate plots (Supplementary materials, Figs. 2 and 5) that the individual moved outside their apparent home range (or did not have central attraction to begin with). Only steps that were outside apparent home ranges without immediate return were classified as dispersal (which is the definition of dispersal; 1). This manual two-way approach to classification resembles both the model-driven NSD-based approach described in [40] and the automated coordinate-based approach in [41]. We also did semivariogram analysis for each individual and these seem to support our NSD analysis (Supplementary materials, Figs. 3 and 6). NSD plots were further used to classify the start and end date of dispersal. To classify stationary steps in between dispersal (temporal home range), we fitted kernel density distribution to the natural logarithm of step speed and selected the minimum value between the two peaks and used it as threshold value (step length of approximately 200 m) for stationary steps, following a method described by [41]. With these steps classified, we then summed the step length from the tracks created for each individual to calculate the distance covered and the speed of each individual in the different movement phases (dispersal phase and stationary phase).

Next, to investigate whether the dispersal steps were made in one particular direction, we converted the step direction from the track of the individual to a cardinal direction using the ‘cimis_degrees_to_compass’ function of the ‘cimir’ package [42]. We then summarised this data as the number of steps taken by each individual in one of the eight cardinal directions during their dispersal phase. The correlation between the number of steps with the directions was then tested using pairwise Spearman’s rank correlations from the ‘psych’ package [43].

#### Effects of movement boundaries on dispersal decisions

To analyze whether movement boundaries affect dispersal decisions by raccoon dogs, we fitted the dispersal data with integrated step-selection functions (iSSFs). We fitted these functions separately to all the individuals, as recommended by Fieberg et al. (2021) with 40 random steps per true step. The dependent variable was the information whether the step was randomly simulated from the observed movement of raccoon dogs or true steps taken by the individuals (conditional logistic regression). Following guidelines in Fieberg et al. (2021) predictor variables were the step length, logarithm of step length, cosine of turn angle and distance of end location of steps from distance rasters to movement boundaries. In addition, interactions between step length, logarithm of step length, cosine of turn angle with distance of start location of steps from boundaries were included in the model to allow the movements to depend on the environment [32]. Step ID was “the strata” i.e. the factor grouping movement in the models.

We created distance rasters for roads (over 3 m wide) and water bodies (rivers over 5 m wide and lakes larger than 22500 hectares). The threshold for lake size was determined by the average and median step lengths of dispersing individuals. We expected that presence of roads or water bodies would decrease (block the movement) or increase (movement rounding the obstacle) step lengths and/or increase angles between the steps (moving along or turning away) compared to those of random steps. To illustrate the data, we present distances of step end locations from the boundaries for cases that the start of step location was close to the boundary (within 100 m), 100–500 m, and > 500 m from the boundary both for raccoon dog steps and for random steps.

After imputation and assessment of quality, we had 22 dispersing individuals from range edge suitable for modelling. 13 individuals of the original 35 dispersing individuals in northern study area were not suitable because of too few steps and uncertain imputation (raw data had too many or too large gaps). From range core we did not have enough data to perform the analysis.

## Results

### Displacement and distance covered by raccoon dogs at range core and range edge

Comparing all individuals, we found that individuals at the range edge (*n* = 62) were displaced over larger distances than individuals at the range core (*n* = 41; t = 4.98, df = 65.24, *p* < 0.001). Individuals at the range edge displaced an average of 32.67 (95% CI: 21.02–44.31) km and the ones at the range core only 3.14 (95% CI: 0.93–5.34) km. Similarly, individuals at the range edge moved larger total distances (t = 2.96, df = 99.09, *p* = 0.004), with the average distance moved by an individual at the range edge being 422.93 (95% CI: 343.56–502.31) km while that of an individual in the range core being 262.48 (95% CI: 187.85–337.11) km (Supplementary materials: Table 1).

The displacement of raccoon dogs at the range edge was not sex dependent (t = 1.84, df = 56.37, *p* = 0.072). However, male raccoon dogs in the range core showed greater displacement distances than females (t = 2.30, df = 23.55, *p* = 0.031; Average displacement, male = 4.03 (95% CI: 1.09–6.98) km, female = 0.74 (95% CI: 0.41–1.08) km). There were two peaks seen in the distance travelled by raccoon dogs, both for the range edge and range core individuals. The peaks in the south were in March and then June-July, whereas in the North these peaks were seen in March-April and July-August (Supplementary materials: Table 2).

### Directionality and movement speed of dispersal at range edge

**Fig. 2:**
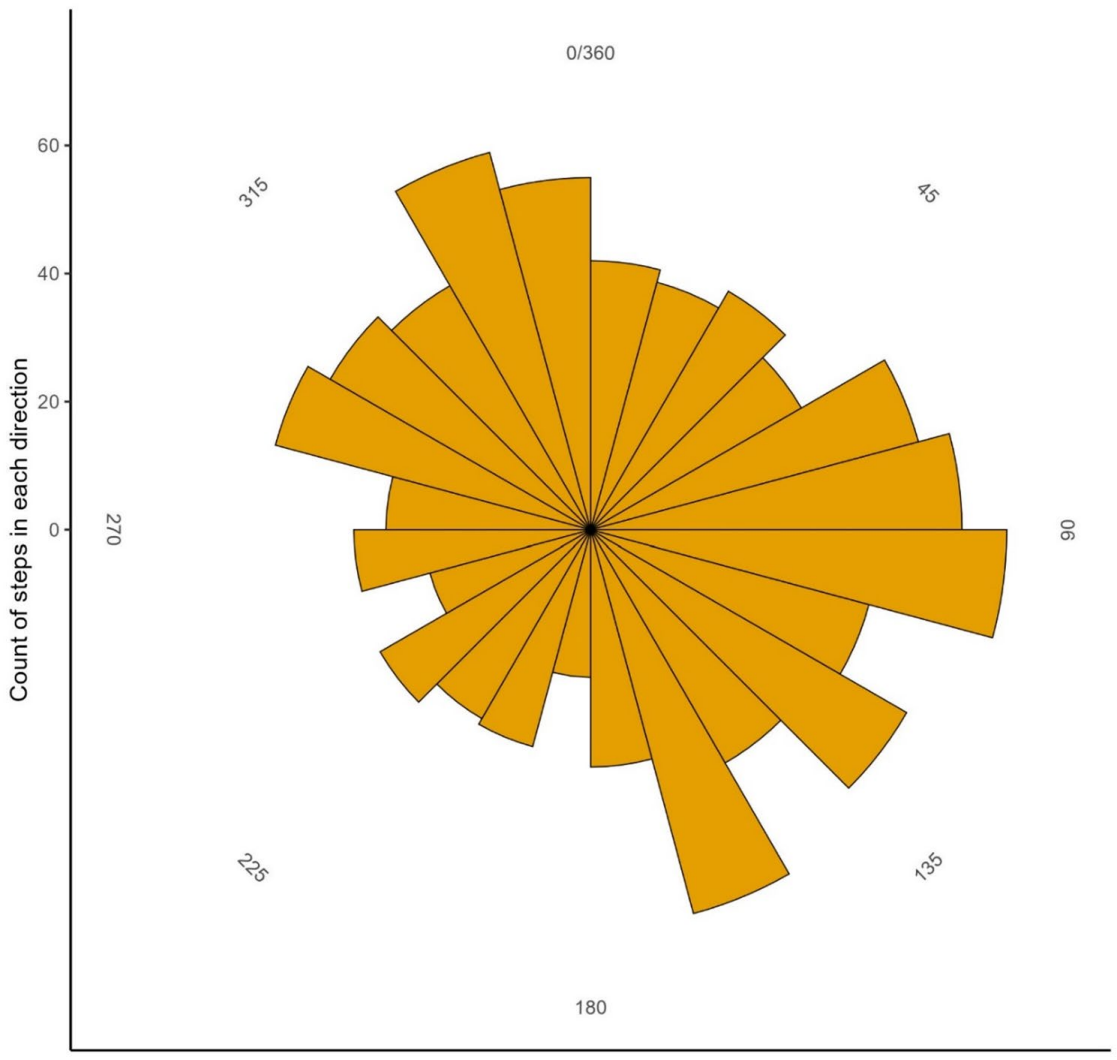
Bar plot of number of steps made by dispersing raccoon dogs (*n* = 22) in different directions by degrees

We did not find that dispersing individuals at the range edge (*n* = 22) moved preferentially in a particular direction (Table 1; Fig. 2). On average the individuals moved with a speed of 0.98 (95%CI: 0.83–0.46) kmph in the dispersal phase and 0.21 (95% CI: 0.17–0.25) kmph during the stationary phase. In Supplementary materials Table 3 we have listed the distances covered during different movement phases and the average speeds during the stationary and dispersal phase of their movement.

**Table 1:** Directionality of dispersing raccoon dogs. Spearman’s correlation between Cardinal directions and the sum of the number of dispersing steps made by 22 dispersing raccoon dogs in that Cardinal direction. None of the correlations were seen to be statistically significant

Direction	No. of steps	Spearman’s correlation
North – North east	125	-0.15
East – North east	149	-0.17
East – South east	167	0.11
South – South east	141	-0.15
South – South west	92	0.10
West – South west	101	-0.07
West – North west	130	0.25
North – North west	160	0.29

### Effects of movement boundaries on dispersal decisions

Based on integrated step-selection analysis we detected no significant or meaningful trends for step lengths and turning angles in relation to waterbodies and roads for individuals classified as dispersing (no or only few individuals showed significant results out of the 22 individuals analyzed; Table 2). Distance (at the end of each step) from waterbodies and roads were quite similar to those of the simulated (random) steps (Table 3). This was the case for all dispersal steps and for those with the start of the step close to waterbodies and roads (Table 3). The distances for simulated locations tended to be slightly larger than observed, but considering the relatively small differences in means and large variance, the difference is not very meaningful. Altogether, these results indicate that the steps raccoon dogs take during dispersal are rather random relative to waterbodies and roads.

**Table 2:** Summary of step selection model results (*n* = 22 GPS tracked dispersing raccoon dogs at the range edge, on average 36.7 ± 21.6 (SD) true steps and 1467 ± 862 random steps per individual)

Term	Coef (mean ± SD)	SE (mean ± SD)	OR (mean ± SD)	Sig. count
Road_end	-0.0002 ± 0.0008	0.0004 ± 0.0004	0.9998 ± 0.0008	0
Water_end	0.0000 ± 0.0003	0.0002 ± 0.0002	1.0000 ± 0.0003	2
Cosine of turning angle	-0.6728 ± 1.0163	0.7318 ± 0.5367	0.7047 ± 0.4791	1
Cosine of turning angle: Road_start	0.0009 ± 0.0021	0.0009 ± 0.0013	1.0009 ± 0.0021	3
Cosine of turning angle: Water_start	0.0002 ± 0.0005	0.0003 ± 0.0003	1.0002 ± 0.0005	0
Log(Step length)	0.3159 ± 0.7898	1.1045 ± 1.4118	2.1051 ± 3.2024	1
Log(Step length): Road_start	0.0010 ± 0.0047	0.0014 ± 0.0032	1.0010 ± 0.0047	0
Log(step length): Water_start	0.0001 ± 0.0009	0.0005 ± 0.0006	1.0001 ± 0.0009	3
Step length	-0.0003 ± 0.0006	0.0006 ± 0.0009	0.9997 ± 0.0006	0
Step length: Road_start	0.0000 ± 0.0000	0.0000 ± 0.0000	1.0000 ± 0.0000	0
Step length: Water_start	0.0000 ± 0.0000	0.0000 ± 0.0000	1.0000 ± 0.0000	1

Coef = coefficient, SE = standard error, OR = odds ratio, Sig. Count = number of individuals with significant P-values at significance level of 0.05 out of the 22 individuals analyzed

**Table 3:** Observed and simulated (random) distances of 22 dispersing adult raccoon dogs from the nearest large waterbody or road

	Observed (average ± SE) in meters	Simulated (average ± SE) in meters
*Step end locations from the nearest large waterbody*		
All locations	2485 ± 97	2534 ± 15
Locations with step start < 500 m from water body	698 ± 65	739 ± 10
Locations with step start < 100 m from water body	545 ± 88	648 ± 14
*Step end locations from the nearest road*		
All locations	1140 ± 42	1180 ± 7
Locations with step start < 500 m from road	610 ± 35	671 ± 6
Locations with step start < 100 m from road	667 ± 72	690 ± 12

Distance measured for the locations at the end of each step. Data for all steps and for steps with the start location of the step being 100–500 m or < 100 m from the nearest waterbody or road

## Discussion

Here, we have studied raccoon dogs in the range core and range edge in Finland. Our results show that raccoon dogs in the range core (Southern Finland) move much shorter distances than those at the range edge (Northern Finland). Of the individuals in the north that show dispersal movements, their dispersal steps were not seen to be directionally north or more in any particular direction. Finally, the dispersing individuals were also not seen to react to movement barriers such as the edges of water bodies or roads or using the barriers to direct their movements.

Earlier research indicates that raccoon dogs in Europe have not reached their full potential of occupying niches and hence the risk of this invasive species spreading further from their current distribution is clear [44]. We show that raccoon dogs at the range edge move and displace longer distances than the ones at the range core. This supports the species’ potential to colonize areas outside the current distribution. We were not able to study what is behind the high movement activity in the range edge, but possible explanations can be speculated on. Firstly, resources are scarcer in the north than in the south due to climatic differences. Hence, it is possible that range edge raccoon dogs must move longer distances than those in the range core to find suitable habitat or to encounter potential mates [45]. Indeed, raccoon dogs are the most common mesopredator in the south [46] of the country. Hence, the lower density of con-specifics could aid longer movements in the range edge. Secondly, the individuals in range edge may have been more active than individuals in range core. This pattern of higher movements at the range edge is seen in earlier studies when the resources and habitat availability have been similar to that in range core [47]. Studies on different taxa have also shown that individuals at the range edge and core show different behaviours [18]. Future research is needed to identify factors behind the long movement distances of raccoon dogs in range edge that will help us further understand the raccoon dog invasion.

The long dispersal distances observed in the north are also notable, because we only studied adult individuals of reproductive age and did not have data on natal dispersal. In mammals, the natal dispersal period is usually the main dispersal period with longer movement distances [1]. In their native range, subadult raccoon dogs are seen to have larger home ranges than adults, supporting higher movement activity for subadults [47]. The natal dispersal in earlier raccoon dog studies is mainly observed in late summer – autumn [48, 49]. A loss of partner can also lead to longer movements in adult raccoon dogs [45]. Many of our study individuals experienced partner removal. This has likely increased the readiness for dispersal in our adult study animals, but the removal of partners is unlikely to explain the lower movement activity observed in range core individuals. Both areas experienced similar eradication programs for mates of GPS-tracked raccoon dogs, but our earlier analysis indicated increased movement distances only in the southern study area and not in the north for a subset of individuals with data on partner removals [30]. Consequently, partner removal seems an unlikely explanation for the difference in movement distances between range core and range edge observed in the current study. We observed two peaks in movement activity before and after the breeding season in the respective latitudes. This is an intuitive result as the adult raccoon dogs would not be moving out of their established territories during the breeding season if they have a litter.

Focusing on the movement of individual raccoon dogs at the range edge, we did not find them to disperse preferentially polewards. There was no apparent directionality in the movements of the dispersing raccoon dogs. In the north, the resources are scarcer and climatic conditions harsher but increasing populations in south may push more individuals towards north. Research on multiple taxa has shown that animals are shifting their home ranges towards the poles [50]. In our case, even though the individual raccoon dogs are not moving towards the north preferentially, 20 individuals (i.e. one-third of our tracked individuals) have been recoded moving above the artic circle which was considered the northern range edge for Finnish raccoon dogs in the 1990s [51]. Movements of raccoon dogs to more northern regions of Finland are concerning as these regions are connected by land to the neighboring countries of Sweden and Norway, which still have low populations of this invasive species [52]. Like earlier work from Swedish populations of range edge individuals [45], we also found that there was no difference in the movement behavior of male and female raccoon dogs at the range edge. However, at the range core we found that male raccoon dogs displaced over larger distances. In general, males in mammals show higher movement distances than females [53]. In our case, the population is heavily managed, and individuals could move longer distances in search of mates if their existing mate is killed [45]. For example, individuals losing a partner may have been selected to be males in our data in range core, which may explain the observed higher movements in males.

Roads and other linear barriers can be used by animals to direct their movements, for example, in larger mammals [7, 8] or they can hinder the movement and dispersal of animals [54]. Here, we show that raccoon dogs’ movements are not limited by encountering movement boundaries such as roads or water bodies. The dispersing individuals at the range edge had similar movements regardless of whether they encountered these boundaries. It is notable that apart from the landscape features explored here, there are no clear barriers to raccoon dog movements in our northern study area. We have, for example, earlier found that raccoon dogs at some level prefer agricultural fields and open bogs [39], which are generally considered barriers to the movement of other forest animals. Earlier research on smaller mammals such as squirrels also showed that roads are not a barrier to their dispersal movements [14, 15]. Hence, like in other species if there are available habitats [15, 55], dispersing raccoon dogs might cross large sized barriers to occupy these habitats. For management of raccoon dogs, our results indicate that concentrating management efforts along linear barriers such as roads and edges of water bodies would not be very effective. There obviously would be a threshold where the barriers become uncrossable. For example, it might be difficult for raccoon dogs to cross the largest rivers, such as Torniojoki river between Finland and Sweden, possibly slowing down the dispersal of raccoon dogs between the countries. However, even this river seemed to be crossed as locations of an individual were observed in both sides of the river (Fig. 1; the river forms the boundary between Sweden and Finland). Based on our earlier analysis on raccoon dogs in southern coastal area the crossing of water areas can be made easier by ice cover [29].

## Conclusions

Our results highlight that the invasive raccoon dogs in Finland are highly active at the range edge and are likely continuing to expand their range. It is easy to underestimate the movements of a species if the movements at the non-equilibrium stage at the range edge are not studied [16]. The reasons behind difference in movement behaviour of the individuals between range core and range edge needs to be studied further as this has consequences for their spread. Here we show that the range edge individuals are more active and do not change their movements in response to movement boundaries such as roads or water bodies, indicating that they could potentially keep dispersing into new territories so far as there is suitable habitat. The high activity shown by the individuals at the range edge is concerning as the invasive species is now moving actively in the highly sensitive northern boreal habitats. A novel mesopredator will impact this ecosystem that is already under immense pressure due to climate change. Raccoon dogs have large home ranges in northern Finland and utilize habitats important for frogs and ground-nesting birds [39] while being capable of eating their eggs, which are especially attractive food items in resource-scarce northern landscape. Boreal habitats are globally important breeding sites for birds that could be adversely affected by the presence of novel predators and hence the behaviour of range edge individuals deserves more attention.

## Supplementary Information

Below is the link to the electronic supplementary material.

Supplementary Material 1

Supplementary Material 2

Supplementary Material 3

Supplementary Material 4

Supplementary Material 5

Supplementary Material 6

Supplementary Material 7

## Data Availability

The movement data of the northern animals is produced and therefore owned by the Finnish wildlife agency, but the authors have permission to use it and publish it in a form of scientific articles but cannot be made available openly. The southern data is owned by the University of Turku and SLSHY ry and is freely available from https://www.movebank.org/cms/webapp?gwt_fragment=page%3Dstudies%2Cpath%3Dstudy4656276921 for individuals in the archipelago and at 10.5281/zenodo.14670904 for inland individuals.
