# Supplementary material for: Northern populations of Finnish raccoon dogs are active at the range edge and unhindered by movement boundaries
Source: Mov Ecol. 2025 Nov 12;13:81. doi: 10.1186/s40462-025-00601-1 (PMC12606842; doi:10.1186/s40462-025-00601-1)
Supplement: Supplementary file 1 — Supplementary Material 1 [file 40462_2025_601_MOESM1_ESM.pdf]

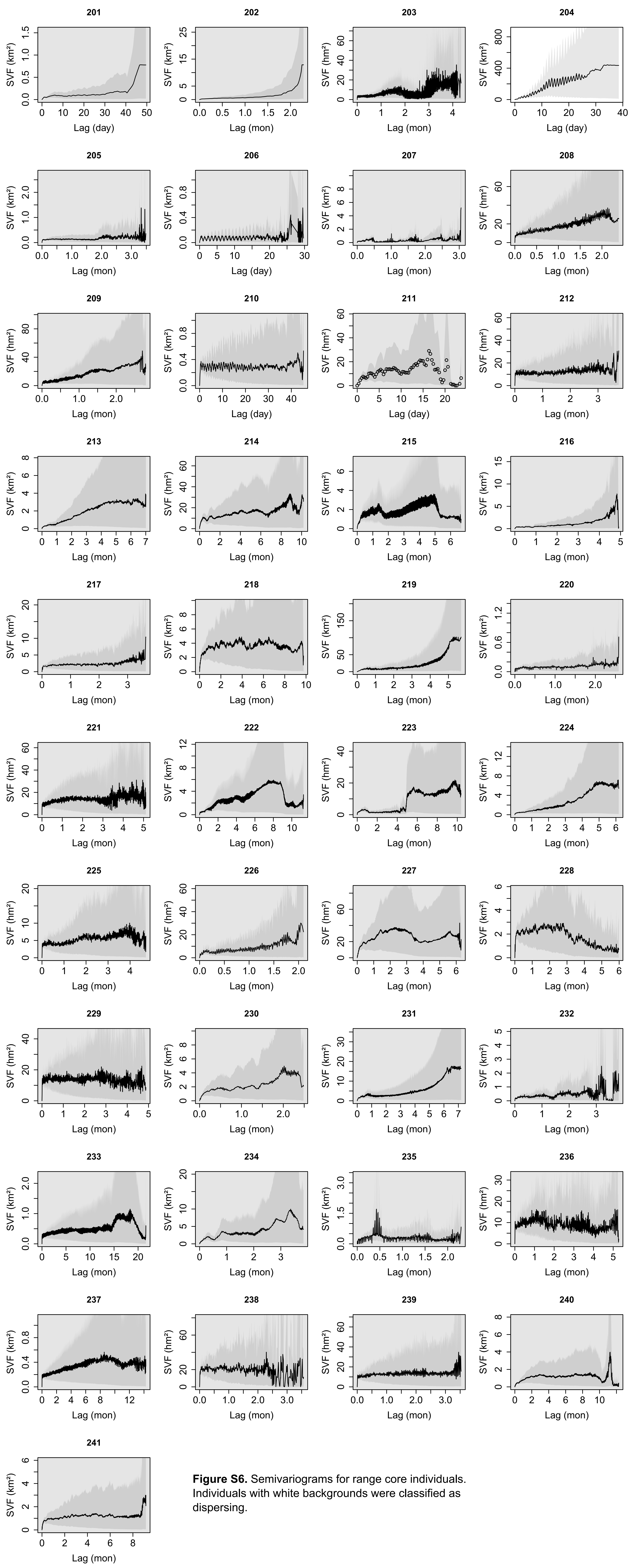

Figure S6. Semivariograms for range core individuals. Individuals with white backgrounds were classified as dispersing.
