# Supplementary material for: Northern populations of Finnish raccoon dogs are active at the range edge and unhindered by movement boundaries
Source: Mov Ecol. 2025 Nov 12;13:81. doi: 10.1186/s40462-025-00601-1 (PMC12606842; doi:10.1186/s40462-025-00601-1)
Supplement: Supplementary file 3 — Supplementary Material 3 [file 40462_2025_601_MOESM3_ESM.pdf]

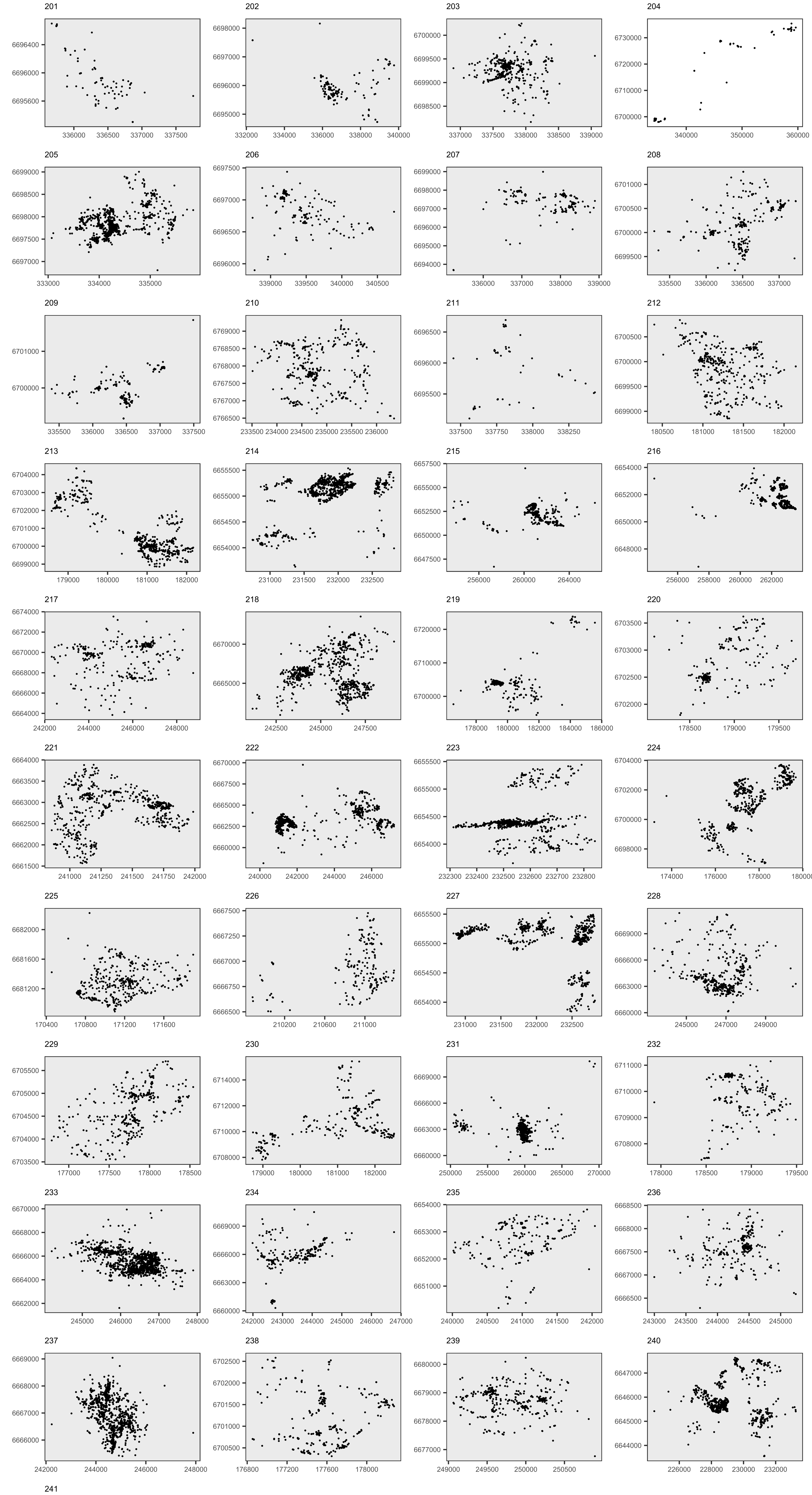

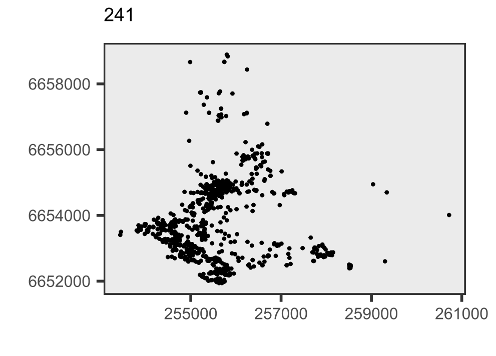

Figure S5. Coordinate plots used for the classification of range core raccoon dogs to dispersing and non-dispersing individuals. Here all the plots with the white background (n = 1) were classified as dispersing. Some outliers were removed to focus the plots. Latitude is on the Y-axis and longitude on the X-axis. Coordinates are on the ETRS-TM35FIN coordinate system.
