# Supplementary figures and images for: Northern populations of Finnish raccoon dogs are active at the range edge and unhindered by movement boundaries
Source: Mov Ecol. 2025 Nov 12;13:81. doi: 10.1186/s40462-025-00601-1 (PMC12606842; doi:10.1186/s40462-025-00601-1)

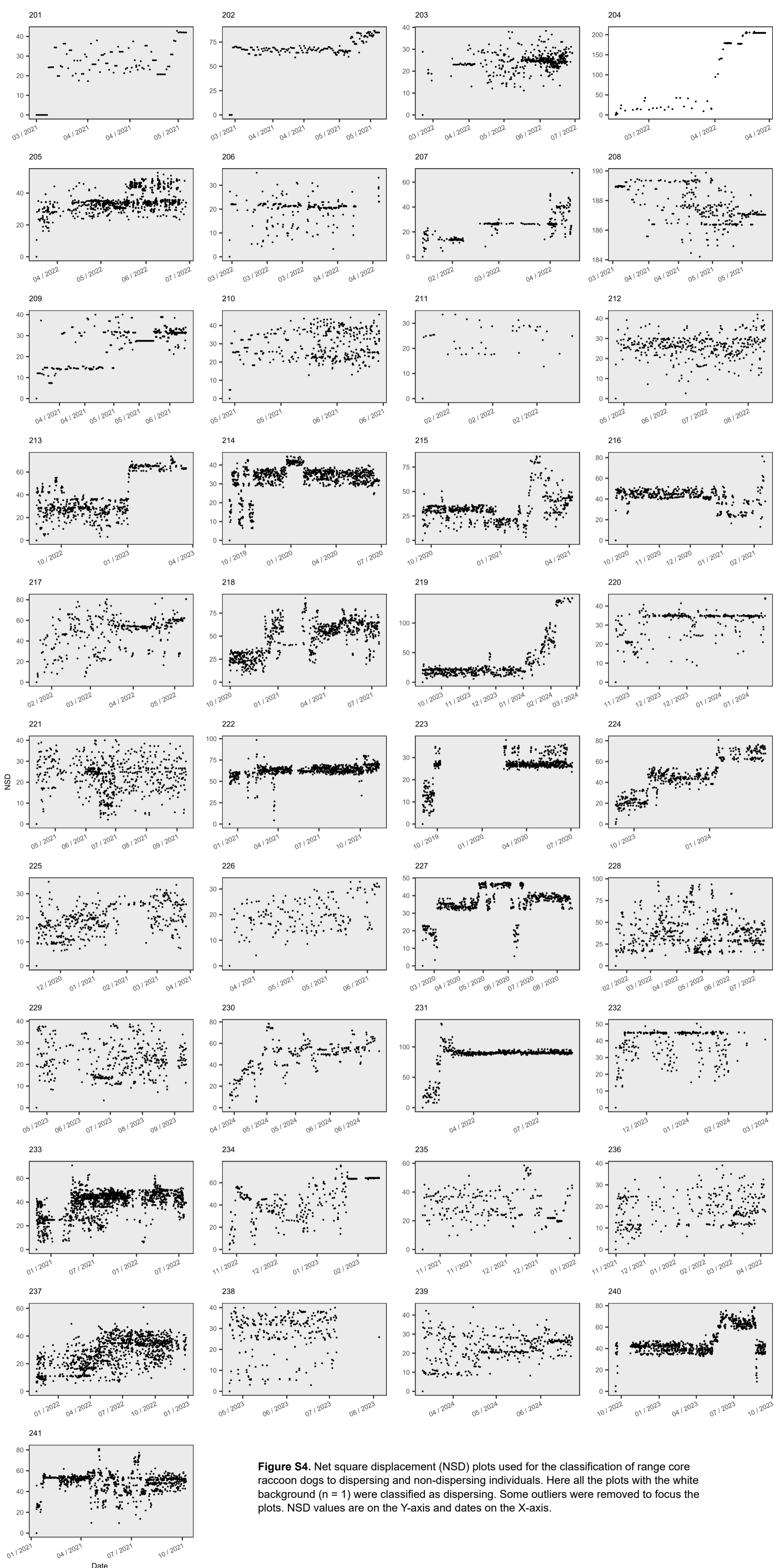

Supplement: Supplementary file 4 — Supplementary Material 4 [file 40462_2025_601_MOESM4_ESM.pdf]
