# Supplementary material for: Northern populations of Finnish raccoon dogs are active at the range edge and unhindered by movement boundaries
Source: Mov Ecol. 2025 Nov 12;13:81. doi: 10.1186/s40462-025-00601-1 (PMC12606842; doi:10.1186/s40462-025-00601-1)
Supplement: Supplementary file 5 — Supplementary Material 5 [file 40462_2025_601_MOESM5_ESM.pdf]

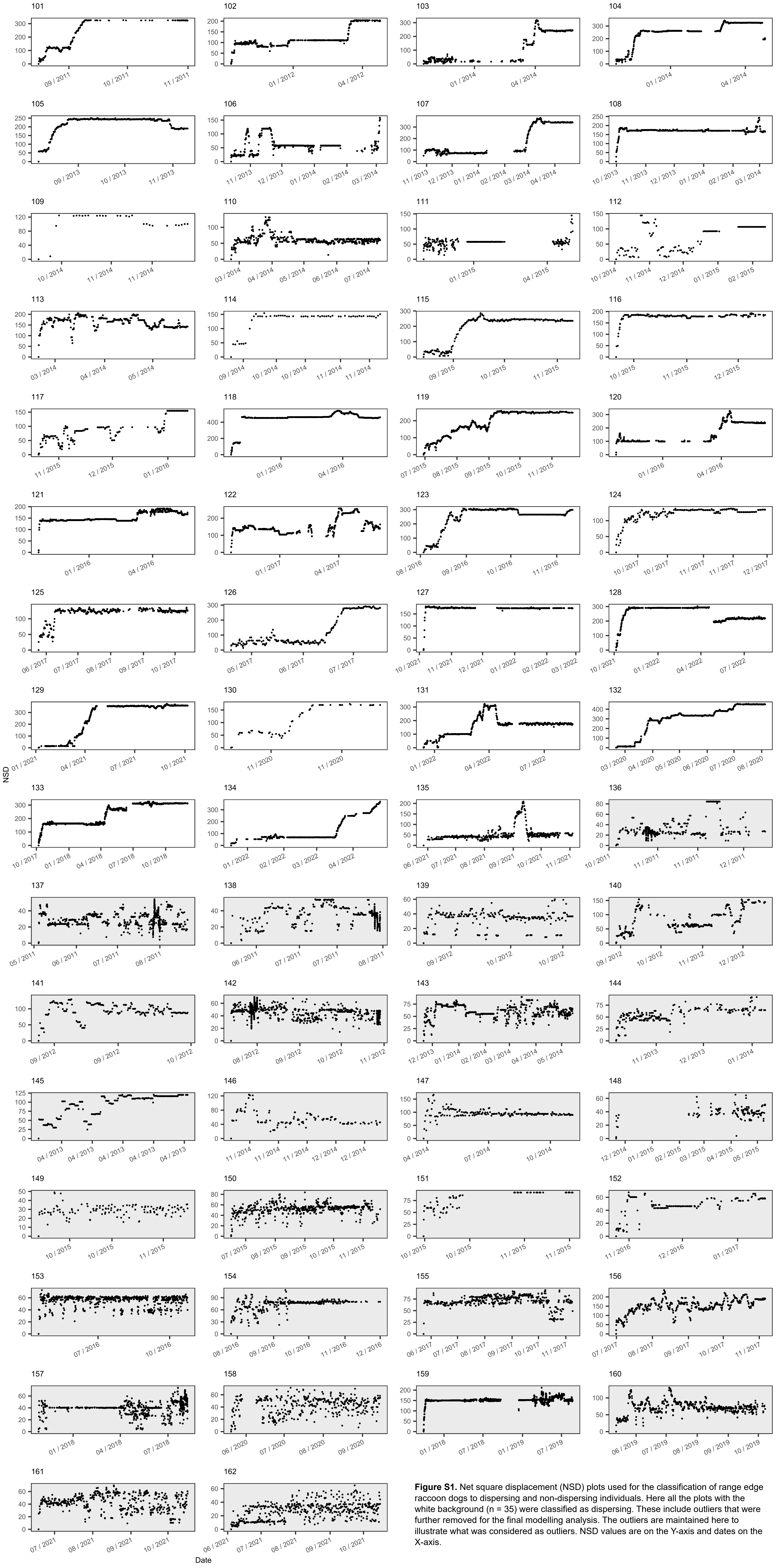

Figure S1. Net square displacement (NSD) plots used for the classification of range edge raccoon dogs to dispersing and non-dispersing individuals. Here all the plots with the white background (n = 35) were classified as dispersing. These include outliers that were further removed for the final modelling analysis. The outliers are maintained here to illustrate what was considered as outliers. NSD values are on the Y-axis and dates on the X-axis.
