# Supplementary material for: Northern populations of Finnish raccoon dogs are active at the range edge and unhindered by movement boundaries
Source: Mov Ecol. 2025 Nov 12;13:81. doi: 10.1186/s40462-025-00601-1 (PMC12606842; doi:10.1186/s40462-025-00601-1)
Supplement: Supplementary file 7 — Supplementary Material 7 [file 40462_2025_601_MOESM7_ESM.docx]

**Supplementary materials**

Table 1: The distances covered, displacement and tracking period of each of the individuals in the study.

| Type of raccoon dog | Individual | Sex | Displacement (km) | Distance (km) | Tracking period |
| --- | --- | --- | --- | --- | --- |
| Range edge | 137 | Male | 0.504428 | 174.9161 | 2011-05-04 - 2011-08-20 |
| Range edge | 138 | Female | 1.08363 | 122.7154 | 2011-06-09 - 2011-07-30 |
| Range edge | 139 | Female | 1.466217 | 105.3758 | 2012-09-08 - 2012-10-17 |
| Range edge | 140 | Male | 20.17336 | 390.2577 | 2012-08-29 - 2012-12-17 |
| Range edge | 141 | Male | 6.837001 | 215.2336 | 2012-08-29 - 2012-09-29 |
| Range edge | 142 | Female | 0.716303 | 439.4606 | 2012-07-13 - 2012-10-29 |
| Range edge | 143 | Male | 3.482757 | 435.4638 | 2013-11-21 - 2014-05-13 |
| Range edge | 103 | Female | 58.98998 | 484.2129 | 2013-10-19 - 2014-05-25 |
| Range edge | 104 | Male | 37.85644 | 486.0221 | 2013-10-28 - 2014-04-23 |
| Range edge | 105 | Female | 34.70191 | 380.6091 | 2013-08-06 - 2013-11-09 |
| Range edge | 144 | Female | 4.335404 | 171.7812 | 2013-10-06 - 2014-01-08 |
| Range edge | 106 | NA | 17.19859 | 228.1633 | 2013-10-13 - 2014-03-03 |
| Range edge | 107 | Male | 111.1903 | 686.6352 | 2013-10-30 - 2014-04-21 |
| Range edge | 145 | Female | 12.11908 | 113.5233 | 2013-03-27 - 2013-04-28 |
| Range edge | 108 | Male | 29.02395 | 439.452 | 2013-10-01 - 2014-03-06 |
| Range edge | 146 | Male | 1.287696 | 123.1287 | 2014-11-06 - 2014-12-10 |
| Range edge | 109 | Male | 6.245274 | 11.64019 | 2014-10-14 - 2014-11-23 |
| Range edge | 147 | Male | 0.265786 | 479.7658 | 2014-03-29 - 2014-10-30 |
| Range edge | 148 | Female | 1.005204 | 140.7209 | 2014-11-21 - 2015-05-08 |
| Range edge | 110 | Male | 3.754117 | 470.73 | 2014-02-22 - 2014-07-11 |
| Range edge | 111 | Male | 18.65416 | 183.796 | 2014-11-01 - 2015-04-30 |
| Range edge | 112 | Male | 11.54518 | 120.6615 | 2014-10-05 - 2015-02-08 |
| Range edge | 113 | Male | 29.35229 | 696.8017 | 2014-02-19 - 2014-05-22 |
| Range edge | 114 | Male | 17.34052 | 75.343 | 2014-09-12 - 2014-11-16 |
| Range edge | 115 | NA | 54.87361 | 413.9973 | 2015-08-15 - 2015-11-08 |
| Range edge | 121 | Female | 19.29543 | 448.1292 | 2015-10-21 - 2016-05-20 |
| Range edge | 116 | Male | 26.89073 | 165.5627 | 2015-09-21 - 2015-12-12 |
| Range edge | 149 | Female | 1.442999 | 95.81277 | 2015-09-21 - 2015-11-07 |
| Range edge | 150 | Male | 1.478426 | 532.2872 | 2015-06-16 - 2015-11-15 |
| Range edge | 151 | Female | 4.930068 | 58.38798 | 2015-09-30 - 2015-11-14 |
| Range edge | 117 | Female | 23.9262 | 154.2793 | 2015-10-21 - 2016-01-11 |
| Range edge | 118 | Male | 211.2087 | 1015.379 | 2015-10-20 - 2016-05-24 |
| Range edge | 119 | Female | 61.53566 | 573.48 | 2015-06-30 - 2015-11-20 |
| Range edge | 120 | Male | 58.31845 | 680.0312 | 2015-10-21- 2016-06-06 |
| Range edge | 152 | Male | 3.371814 | 46.32562 | 2016-10-25 - 2017-01-15 |
| Range edge | 122 | Male | 21.34866 | 699.6725 | 2016-10-19 - 2017-06-01 |
| Range edge | 153 | Female | 2.46652 | 393.7801 | 2016-04-16 - 2016-10-23 |
| Range edge | 123 | Male | 88.8823 | 608.4765 | 2016-08-04 -2016-11-10 |
| Range edge | 154 | Male | 7.341419 | 318.2128 | 2016-07-27 - 2016-11-14 |
| Range edge | 155 | Female | 0.783317 | 541.2064 | 2017-05-24 - 2017-11-06 |
| Range edge | 156 | Male | 40.70434 | 1176.913 | 2017-07-01 - 2017-11-05 |
| Range edge | 124 | Female | 15.71011 | 178.2277 | 2017-09-21 - 2017-11-28 |
| Range edge | 125 | Female | 17.78197 | 437.3536 | 2017-05-25 -2017-10-12 |
| Range edge | 126 | Male | 75.41787 | 382.9573 | 2017-04-19 - 2017-07-15 |
| Range edge | 157 | Male | 2.694657 | 419.4566 | 2017-10-27 - 2018-08-07 |
| Range edge | 131 | Male | 29.24571 | 844.4625 | 2021-12-15 - 2022-08-15 |
| Range edge | 132 | Male | 202.973 | 765.4016 | 2020-02-20 - 2020-08-03 |
| Range edge | 133 | Female | 98.5102 | 1344.15 | 2017-10-07 - 2018-11-30 |
| Range edge | 134 | Female | 121.8102 | 267.2429 | 2021-12-18 - 2022-04-22 |
| Range edge | 162 | Female | 1.404407 | 394.6743 | 2021-06-04 - 2021-10-14 |
| Range edge | 135 | Female | 1.451085 | 447.4512 | 2021-06-02 - 2021-11-02 |
| Range edge | 158 | Male | 3.079151 | 483.7271 | 2020-05-20 - 2020-09-13 |
| Range edge | 127 | Male | 23.48033 | 205.1 | 2021-10-05 - 2022-02-25 |
| Range edge | 128 | Male | 46.03347 | 906.9743 | 2021-10-05 - 2022-08-12 |
| Range edge | 159 | Male | 23.57328 | 1398.82 | 2017-09-28 - 2019-08-20 |
| Range edge | 160 | Female | 1.98732 | 601.9356 | 2019-05-12 - 2019-10-05 |
| Range edge | 129 | Male | 129.1227 | 938.7687 | 2021-01-11 - 2021-10-04 |
| Range edge | 161 | Male | 1.963211 | 318.4401 | 2021-06-18 - 2021-10-15 |
| Range edge | 130 | Female | 25.48349 | 67.50224 | 2020-11-05 - 2020-11-18 |
| Range edge | 101 | Male | 103.9215 | 264.3866 | 2011-08-17 - 2011-10-31 |
| Range edge | 136 | Female | 0.713005 | 143.908 | 2011-10-17 - 2011-12-02 |
| Range edge | 102 | Male | 41.08688 | 312.6097 | 2011-10-12 - 2012-04-21 |
| Range core | 212 | Female | 0.851454 | 185.8032 | 2022-04-26 - 2022-08-12 |
| Range core | 226 | Male | 0.749046 | 50.16433 | 2021-04-06 - 2021-06-04 |
| Range core | 228 | Male | 0.387168 | 570.289 | 2022-01-19 - 2022-07-13 |
| Range core | 231 | Male | 7.666186 | 494.878 | 2022-01-21 - 2022-08-18 |
| Range core | 233 | Male | 0.4223 | 1096.746 | 2020-11-01 - 2022-07-31 |
| Range core | 234 | Male | 4.153534 | 169.4204 | 2022-10-27 - 2023-02-16 |
| Range core | 235 | Male | 0.773056 | 148.0524 | 2021-10-24 - 2021-12-28 |
| Range core | 236 | Female | 0.740068 | 149.8968 | 2021-11-01 - 2022-04-03 |
| Range core | 237 | Male | 1.482929 | 790.2294 | 2021-11-01 - 2022-12-24 |
| Range core | 238 | Female | 0.297907 | 134.7507 | 2023-04-22 - 2023-07-05 |
| Range core | 213 | Male | 3.074787 | 305.4851 | 2022-08-28 - 2023-03-21 |
| Range core | 240 | Male | 0.06103 | 378.205 | 2022-09-18 - 2023-09-14 |
| Range core | 241 | Male | 2.332109 | 503.9787 | 2021-01-11 - 2021-10-05 |
| Range core | 214 | Female | 1.106854 | 249.0415 | 2019-08-30 - 2020-06-26 |
| Range core | 219 | Male | 19.37211 | 285.4846 | 2023-09-10 - 2024-02-20 |
| Range core | 220 | Male | 0.313464 | 89.23282 | 2023-11-09 - 2024-01-22 |
| Range core | 223 | Female | 0.826067 | 91.8521 | 2019-09-01 - 2020-07-02 |
| Range core | 224 | Male | 5.550077 | 314.9832 | 2023-09-09 - 2024-03-07 |
| Range core | 227 | Female | 0.884395 | 126.0714 | 2020-02-16 - 2020-08-16 |
| Range core | 215 | Female | 1.974172 | 352.8703 | 2020-09-20 - 2021-04-03 |
| Range core | 229 | Male | 0.121324 | 202.867 | 2023-04-21 - 2023-09-11 |
| Range core | 230 | Male | 4.335631 | 147.8849 | 2024-04-13 - 2024-06-21 |
| Range core | 232 | Male | 2.826311 | 139.0229 | 2023-11-08 - 2024-02-08 |
| Range core | 239 | Female | 0.121324 | 202.867 | 2023-04-21 - 2023-09-11 |
| Range core | 216 | Male | 5.423195 | 188.7817 | 2020-09-20 - 2021-02-09 |
| Range core | 217 | Male | 3.866796 | 468.2236 | 2022-01-22 - 2022-05-07 |
| Range core | 218 | Male | 2.863665 | 778.148 | 2020-09-30 - 2021-07-14 |
| Range core | 221 | Female | 0.61883 | 310.7592 | 2021-04-12 - 2021-09-09 |
| Range core | 222 | Male | 2.057491 | 619.1095 | 2020-12-14 - 2021-11-10 |
| Range core | 225 | Female | 0.463786 | 138.6467 | 2020-11-09 -2021-03-26 |
| Range core | 201 | Female | 1.779233 | 30.84241 | 2021-03-16 - 2021-05-01 |
| Range core | 210 | Female | 0.247432 | 178.684 | 2021-04-29 - 2021-06-13 |
| Range core | 211 | Male | 0.554531 | 15.41808 | 2022-02-11- 2022-02-28 |
| Range core | 202 | Male | 3.202657 | 67.68854 | 2021-03-14 - 2021-05-17 |
| Range core | 203 | Female | 1.086323 | 125.8792 | 2022-02-24 - 2022-06-26 |
| Range core | 204 | Male | 41.78015 | 86.62069 | 2022-03-06 -2022-04-13 |
| Range core | 205 | Female | 0.713962 | 279.1118 | 2022-03-18 -2022-06-27 |
| Range core | 206 | Female | 1.031654 | 59.81982 | 2022-03-13 - 2022-04-12 |
| Range core | 207 | Female | 0.671868 | 64.20803 | 2022-01-14 - 2022-04-13 |
| Range core | 208 | Male | 1.003949 | 108.7097 | 2021-03-16 - 2021-05-24 |
| Range core | 209 | Female | 0.903658 | 60.78105 | 2021-03-20 - 2021-06-09 |

Table 2: Summary of the number of individuals sampled in the range core and range edge for each month of the years and distances covered per individual at the range core and range edge across the year.

| Month | Type of raccoon dog | No of. Individuals sampled | Distance/ no. Of individuals (km) |
| --- | --- | --- | --- |
| 1 | Range edge | 24 | 14.972 |
| 1 | Range core | 21 | 32.372 |
| 2 | Range edge | 25 | 28.023 |
| 2 | Range core | 24 | 41.300 |
| 3 | Range edge | 24 | 123.940 |
| 3 | Range core | 26 | 50.700 |
| 4 | Range edge | 25 | 143.955 |
| 4 | Range core | 31 | 39.537 |
| 5 | Range edge | 24 | 79.048 |
| 5 | Range core | 26 | 48.107 |
| 6 | Range edge | 24 | 75.869 |
| 6 | Range core | 22 | 51.800 |
| 7 | Range edge | 25 | 98.768 |
| 7 | Range core | 15 | 56.087 |
| 8 | Range edge | 28 | 110.026 |
| 8 | Range core | 13 | 48.103 |
| 9 | Range edge | 29 | 109.089 |
| 9 | Range core | 16 | 38.116 |
| 10 | Range edge | 46 | 65.524 |
| 10 | Range core | 15 | 41.238 |
| 11 | Range edge | 41 | 60.240 |
| 11 | Range core | 17 | 46.590 |
| 12 | Range edge | 27 | 24.930 |
| 12 | Range core | 17 | 39.364 |

Table 3: Distances covered by dispersing raccoon dogs at the range edge during their dispersal and stationary phases.

| ID | Class | Distance (km) | Average speed (kmph) | No. of steps |
| --- | --- | --- | --- | --- |
| 101 | Dispersal | 83.3232 | 1.02847 | 27 |
| 101 | Stationary | 68.6731 | 0.19879 | 115 |
| 102 | Dispersal | 49.0735 | 0.74335 | 22 |
| 102 | Stationary | 165.898 | 0.11073 | 523 |
| 103 | Dispersal | 171.072 | 1.58425 | 36 |
| 103 | Stationary | 304.01 | 0.15378 | 659 |
| 104 | Dispersal | 176.703 | 0.78546 | 75 |
| 104 | Stationary | 181.915 | 0.07735 | 784 |
| 105 | Dispersal | 89.7542 | 0.55421 | 54 |
| 105 | Stationary | 315.954 | 0.177 | 595 |
| 107 | Dispersal | 273.179 | 1.13833 | 80 |
| 107 | Stationary | 393.609 | 0.15472 | 848 |
| 108 | Dispersal | 147.016 | 1.1668 | 42 |
| 108 | Stationary | 252.327 | 0.13857 | 607 |
| 119 | Dispersal | 87.6099 | 0.46589 | 66 |
| 119 | Stationary | 183.382 | 0.31615 | 221 |
| 120 | Dispersal | 212.398 | 1.49576 | 53 |
| 120 | Stationary | 111.883 | 0.23805 | 173 |
| 121 | Dispersal | 43.0593 | 0.97862 | 16 |
| 121 | Stationary | 150.132 | 0.14747 | 379 |
| 122 | Dispersal | 251.722 | 0.96804 | 93 |
| 122 | Stationary | 99.3373 | 0.17366 | 213 |
| 123 | Dispersal | 123.516 | 1.28662 | 35 |
| 123 | Stationary | 135.153 | 0.39978 | 126 |
| 124 | Dispersal | 51.0705 | 0.50069 | 38 |
| 124 | Stationary | 33.2324 | 0.12041 | 103 |
| 125 | Dispersal | 32.0581 | 0.66741 | 17 |
| 125 | Stationary | 119.333 | 0.36831 | 121 |
| 126 | Dispersal | 25.494 | 0.84933 | 10 |
| 126 | Stationary | 147.144 | 0.38119 | 144 |
| 128 | Dispersal | 92.4646 | 0.69003 | 48 |
| 128 | Stationary | 112.945 | 0.11718 | 359 |
| 129 | Dispersal | 145.293 | 0.90808 | 58 |
| 129 | Stationary | 311.616 | 0.33219 | 351 |
| 131 | Dispersal | 271.037 | 1.30306 | 77 |
| 131 | Stationary | 332.925 | 0.25694 | 483 |
| 132 | Dispersal | 300.612 | 1.22724 | 86 |
| 132 | Stationary | 135.693 | 0.21215 | 240 |
| 133 | Dispersal | 160.697 | 0.93437 | 63 |
| 133 | Stationary | 714.291 | 0.27963 | 954 |
| 134 | Dispersal | 127.933 | 1.16496 | 38 |
| 134 | Stationary | 56.5669 | 0.09897 | 214 |
| 135 | Dispersal | 65.4315 | 0.77864 | 31 |
| 135 | Stationary | 129.557 | 0.26247 | 180 |

Figure 1: Net square displacement (NSD) plots used for the classification of range edge raccoon dogs to dispersing and non-dispersing individuals. Here all the plots with the white background (n = 35) were classified as dispersing. These include outliers that were further removed for the final modelling analysis. The outliers are maintained here to illustrate what was considered as outliers. NSD values are on the Y-axis and dates on the X-axis.

Figure 2: Coordinate plots used for the classification of range edge raccoon dogs to dispersing and non-dispersing individuals. Here all the plots with the white background (n = 35) were classified as dispersing. These include outliers that were further removed for the final modelling analysis. The outliers are maintained here to illustrate what was considered as outliers. Latitude is on the Y-axis and longitude on the X-axis. Coordinates are on the ETRS-TM35FIN coordinate system.

Figure 3: Semivariograms for range edge individuals. Individuals with white backgrounds were classified as dispersing.

Figure 4: Net square displacement (NSD) plots used for the classification of range core raccoon dogs to dispersing and non-dispersing individuals. Here all the plots with the white background (n = 1) were classified as dispersing. Some outliers were removed to focus the plots. NSD values are on the Y-axis and dates on the X-axis.

Figure 5: Coordinate plots used for the classification of range core raccoon dogs to dispersing and non-dispersing individuals. Here all the plots with the white background (n = 1) were classified as dispersing. Some outliers were removed to focus the plots. Latitude is on the Y-axis and longitude on the X-axis. Coordinates are on the ETRS-TM35FIN coordinate system.

Figure 6: Semivariograms for range core individuals. Individuals with white backgrounds were classified as dispersing.
